# Digital media use and its effects on digital eye strain and sleep quality in adolescents: A new emerging epidemic?

**DOI:** 10.1371/journal.pone.0314390

**Published:** 2024-12-19

**Authors:** Merve Şambel Aykutlu, Hasan Cem Aykutlu, Mehmet Özveren, Rüveyde Garip

**Affiliations:** 1 Department of Ophthalmology, Edirne Sultan 1st Murat State Hospital, Edirne, Turkey; 2 Department of Child and Adolescent Psychiatry, Trakya University Faculty of Medicine, Edirne, Turkey; 3 Department of Ophthalmology, Trakya University Faculty of Medicine, Edirne, Turkey; Bursa Ali Osman Sonmez Oncology Hospital, TÜRKIYE

## Abstract

This study examines the association between excessive digital media use and adverse health outcomes, specifically Digital Eye Strain (DES) and Poor Sleep Quality (PSQ), in adolescents. A cross-sectional survey of 512 participants (aged 11–18 years) assessed DES and PSQ using the Computer Vision Syndrome Questionnaire and the Pittsburgh Sleep Quality Index. We found a high prevalence of DES (63.7%) and PSQ (51.2%). Factors associated with DES included using digital media for more than two hours daily, increased post-pandemic digital consumption, shorter breaks, and PSQ. Extended daily digital media use (>4 hours), bedtime usage, older age, female sex, online education and DES were significantly associated with PSQ. These findings highlight the harmful effects of excessive digital media use on adolescent health, especially post-COVID-19. The intricate link between DES and PSQ underscores the need for public health interventions to promote healthy digital habits.

## Introduction

Digital media have become indispensable companions for adolescents, particularly during the COVID-19 pandemic. With the closure of schools and workplaces, reliance on digital devices for education, work, and entertainment has surged, leading to increased screen time. This shift, initially necessitated by the lack of outdoor activities, has persisted even as pandemic restrictions eased, establishing a new normal of digital lifestyle [1, 2].

However, the pervasive use of digital devices poses significant health risks, particularly to vision and sleep. Digital Eye Strain (DES), or “computer vision syndrome,” is characterized by symptoms such as blurred vision, headaches, and eye discomfort, primarily affecting individuals who use digital devices for prolonged periods [3]. A systematic review and meta-analysis reported DES prevalence ranging from 12% to 99%, with an average of 66% [4]. Prevalence is notably higher among adolescents using digital devices for more than two hours daily [5]. This issue is global, affecting diverse demographics across North America, Europe, Asia, and beyond, with a notable increase to 74% during the pandemic [6].

In the United States, 50% to 90% of school-aged children and teenagers experience inadequate sleep, likely due to extensive use of screen-based media [7]. Devices like computers and smartphones emit blue light from LEDs, which disrupts the circadian rhythm by suppressing melatonin production, thus affecting sleep quality [8, 9]. Extended digital device use was linked to poor sleep quantity and quality, with adolescents who frequently use phones exhibiting later wake-up times and shorter sleep durations [10–12]. Furthermore, daily screen time exceeding two hours was associated with an increased risk of sleep onset difficulties by 20% [13].

The consequences of DES and sleep disturbances extend beyond discomfort, contributing to higher obesity risk, poorer psychological well-being, and cognitive impairments in adolescents [7]. Disrupted sleep was associated with reduced academic performance, impaired concentration, and increased emotional distress [9, 14]. High screen time correlates with shorter sleep durations and daytime fatigue, while internet use was linked to delayed bedtimes and reduced total sleep time [15, 16]. Additionally, DES was associated with musculoskeletal problems like neck pain and increased levels of anxiety and depression, with stress partially mediating this relationship [17, 18].

While DES and poor sleep quality (PSQ) are distinct clinical conditions, they share important common risk factors, particularly prolonged use of digital devices [4, 11]. Moreover, there exists a potential, underexplored link between the two that merits further investigation. For instance, DES can result in various ocular discomfort symptoms such as eye strain, dryness, and headaches, which may interfere with the ability to fall asleep or maintain quality sleep [19, 20]. Conversely, PSQ has been associated with increased dry eye symptoms and reduced tear film stability, potentially exacerbating DES symptoms the following day [21]. This bidirectional relationship suggests a complex interplay between ocular comfort and sleep quality.

The role of blue light emitted by digital screens further supports this interconnection. Blue light exposure, especially in the evening hours, can disrupt circadian rhythms by suppressing melatonin production, thereby affecting sleep onset and quality [8]. Importantly, blue light has also been shown to contribute to visual fatigue and discomfort when viewing digital content potentially through mechanisms such as increased eye dryness and reduced visual performance [22]. The dual impact of blue light on both sleep patterns and visual comfort underscores the intricate relationship between digital device use, DES, and sleep quality.

Additionally, the cognitive and physiological arousal associated with engaging digital content, particularly before bedtime, may contribute to both DES and PSQ. Extended periods of focus on digital screens can lead to reduced blink rate and incomplete blinking, exacerbating dry eye symptoms [23]. Simultaneously, this heightened cognitive engagement can make it difficult to "wind down" for sleep, potentially leading to delayed sleep onset and reduced sleep quality [24]. The combined effects of blue light exposure and cognitive arousal create a complex web of factors that can simultaneously impact both ocular comfort and sleep patterns, further emphasizing the potential interrelationship between DES and PSQ in the context of digital device use.

Despite both DES and PSQ being well-studied in the literature, their co-occurrence and shared risk factors remain understudied, particularly in adolescents whose digital media consumption habits have dramatically changed post-pandemic [25]. This gap in the literature is particularly concerning given the potential for these conditions to compound each other, creating a cycle of discomfort and poor sleep that could significantly impact overall well-being and daily functioning. This study aims to investigate the prevalence of DES and sleep problems among adolescents, exploring their interconnectedness and identifying potential risk factors. By elucidating the relationship between these conditions, we hope to inform more comprehensive strategies for promoting digital well-being and healthy sleep habits among adolescents in our increasingly digitalized world.

## Material and methods

### Participants

This cross-sectional study collected data via questionnaires administered to adolescents (aged 11–18 years) attending the ophthalmology outpatient clinic between November 22, 2021, and June 3, 2022, in Edirne, Turkey. The presence of strabismus, amblyopia, or organic ocular diseases were established as exclusion criteria.

The sample size for our study was determined using a proportion-based sample size calculation formula: $Sample\;size=\frac{\frac{z^{2}\times p(1-p)}{e^{2}}}{1+(\frac{z^{2}\times p(1-p)}{e^{2}N})}$. In this formula, the z-score represents the number of standard deviations from the mean in a normal distribution and is set at 1.96 for a 95% confidence interval. The population proportion (p) is estimated at 0.5 when the actual proportion is unknown, to maximize the sample size. The margin of error (e) is set at 5%, indicating the allowable deviation in the sample’s results from the true population value [26]. Based on the 2020 data from Türkiye İstatistik Kurumu, the population of 10–19 year olds in Edirne was 43202 [27]. Using these parameters, we calculated that our sample size needed to be at least 384 individuals to accurately represent the population, assuming a simple random sampling method without replacement where the sample size is less than 5% of the population.

### Instrumentation

The questionnaire was divided into three sections: Predisposing factors survey developed by the authors, Computer Vision Syndrome Questionnaire (CVS-Q), and Pittsburgh Sleep Quality Index (PSQI).

The survey on predisposing factors examined age, gender, and variables related to digital media use habits. These variables included the type of digital device used (desktop, laptop, tablet, smartphone), the amount of time spent using digital media per day (<2 hours, 2–4 hours, >4 hours), breaks taken during digital media use (yes/no), the break duration (<20 minutes, ≥20 minutes) and frequency of these breaks (every 30 minutes/ 1 hour/ 2 hours), attendance to online education (yes/no), digital media time has increased compared to the pre-pandemic period (yes/no), the preferred timing of digital media use (day/night), and habitual use of digital media 1 hour before bedtime (yes/no). The categorical limits were determined based on values that have been found to be significant in previous studies [5, 13]. Participants completed the questions based on their usage over the last month.

The degree of DES symptoms was assessed using a pre-validated CVS-Q developed by del Mar Seguí et al. [28]. The CVS-Q has demonstrated good psychometric properties, with an internal consistency of Cronbach’s α = 0.78 and test-retest reliability of ICC = 0.802 in its original validation study. It has also shown good validity with a sensitivity of 75% and specificity of 70% [28]. The CVS-Q assess 16 eye strain-related symptoms, such as burning, itching, watering, excessive blinking, redness, pain, heavy eyelids, dryness, blurry vision, double vision, difficulty in near vision, intolerance to light, colored halos, worsening of vision, and headache. The frequency and intensity of these 16 symptoms are used to grade DES. Frequency is graded as follows: never (0 points), occasionally (1 points) (once a week, irregular episodes), and always (2 points) (more than 2–3 times a week). The intensity is scored as moderate (1 points) and intense (2 points). (Frequency X Intensity) produced the following recorded results: 0 = 0; 1 or 2 = 1; and 4 = 2. A CVS-Q score of 6 or above indicate DES. In our study, the CVS-Q showed excellent internal consistency with a Cronbach’s α of 0.9.

PSQI is a widely used self-report scale developed by Buysse et al. [29], which evaluates sleep quality and disorder over a one-month period. A systematic review by Mollayeva et al. [30], analyzing 45 studies across diverse populations and languages, confirmed the PSQI’s robust psychometric properties, reliability, and validity in both clinical and non-clinical settings. The original validation reported good internal consistency (Cronbach’s α = 0.83) and test-retest reliability [29]. The PSQI consists of seven components: subjective sleep quality, sleep latency, sleep duration, habitual sleep efficiency, sleep disturbance, use of sleep medicines, and daytime dysfunction. Each component is scored from 0 to 3 points. The sum of the scores of the seven components gives the total score of the scale, and the scoring varies between 0 and 21. A total score which is greater than 5 means PSQ. In our study, the PSQI showed acceptable internal consistency with a Cronbach’s α of 0.7.

### Data analysis

Data analysis was conducted utilizing SPSS software (version 22.0). We presented descriptive statistics in the form of frequencies and percentages. To examine the relationships and differences within the data, we employed the Chi-Square Test and logistic regression analyses. To adjust for the risk of type I errors due to multiple comparisons, a Bonferroni correction was applied. Criteria for statistical significance were set at a p-value of less than 0.05 and results were reported alongside a 95% confidence interval (CI) to ensure precision in our findings.

### Ethics approval

All participants and their families were provided with detailed information about the study and the written consents were then obtained. All study protocols complied with the principles of the Declaration of Helsinki. Approval for the study was obtained from Trakya University Faculty of Medicine Scientific Research Ethics Committee (2021/323).

## Results

### Demography

The study included 512 individuals with complete data. Table 1 details participant demographics, including gender, age, and digital media usage patterns. The mean age of the participants was 14.5 ± 2.0 years, with 50.6% in early adolescence (11–14 years) and 49.4% in mid to late adolescence (15–18 years). Additionally, 58.2% of the participants were female.

**Table 1:** Distribution of age, sex, pattern of gadget use and their relationship with Digital Eye Strain and poor sleep quality.

Variable	n (%)	DES (%)	p	Poor sleep quality (%)	p
**Age**			**0.029**		
*Early adolescence (11–14)*	259(50.6)	59.1		38.6	**<0.001**
*Mid to late adolescence (15–18)*	253(49.4)	68.4		64.0	
**Sex**			**0.014**		**<0.001**
*Girl*	298(58.2)	68.1		60.1	
*Boy*	214(41.8)	57.5		38.8	
**Preferred digital device**			0.262		0.268
*Desktop*	61(11.9)	54.1		44.3	
*Laptop*	59(11.5)	67.8		52.5	
*Tablet*	27(5.3)	55.6		37.0	
*Smartphone*	365(71.3)	65.2		53.2	
**Daily digital media usage**			**<0.001**		**0.001**
*<2 hours*	40(7.8)	30.0^*a*^		42.5^a^	
*2–4 hours*	208(40.6)	60.6^*b*^		42.3^a^	
*>4 hours*	264(51.6)	71.2^*c*^		59.5^b^	
**Breaks during digital media use**			0.222		0.144
*Yes*	389(76.0)	62.2		49.4	
*No*	123(24.0)	68.3		56.9	
**Break frequency**			0.582		0.361
*Never*	123(24.0)	68.3		56.9	
*Once every >2 hours*	63(12.3)	63.5		54.0	
*Once an hour*	190(37.1)	60.5		50.0	
*Once every 30 min*	136(26.6)	64.0		46.3	
*Break duration*			**<0.001**		0.226
*<20 min*	382(74.6)	68.3		49.7	
≥*20 min*	130(25.4)	50.0		55.4	
**Preferred timing for digital media use**			0.504		0.001
*Daytime*	246(48.0)	62.2		43.5	
*Night-time*	266(52.0)	65.0		58.3	
**Digital media usage 1 hour before bedtime**			**<0.001**		**<0.001**
*Yes*	431(84.2)	67.3		55.0	
*No*	81(15.8)	44.4		30.9	
**Has digital media consumption increased after the pandemic?**			**<0.001**		0.263
*Yes*	370(72.3)	68.4		52.7	
*No*	142(27.7)	51.4		47.2	
**Increased digital media time after pandemic**			**<0.001**		0.247
*None*	142(27.7)	51.4^*a*^		47.2	
*1 hour*	54(10.5)	38.9^*a*^		46.3	
*1–2 hours*	130(25.4)	72.3^*b*^		49.2	
*>2 hours*	186(36.3)	74.2^*b*^		57.0	
**Online education**			0.089		**0.028**
*Yes*	100(19.5)	71		61.0	
*No*	412(80.5)	61.9		48.8	
**DES**			n/a		**<0.001**
*Yes*	326(63.7)	100		62.9	
*No*	186(36.3)	0		30.6	
**Poor sleep quality**			**<0.001**		n/a
*Yes*	262(51.2)	78.2		100	
*No*	250(48.8)	48.4		0	

DES: digital eye strain; SD: standart deviation.

*Note*: Table based on Chi-Square test analysis data. Bold numbers indicate the statistically significant differences. Different letters (^a^,^b^) indicate the statistically significant group differences by bonferroni correction.

### The pattern of digital device use

**Fig 1:**
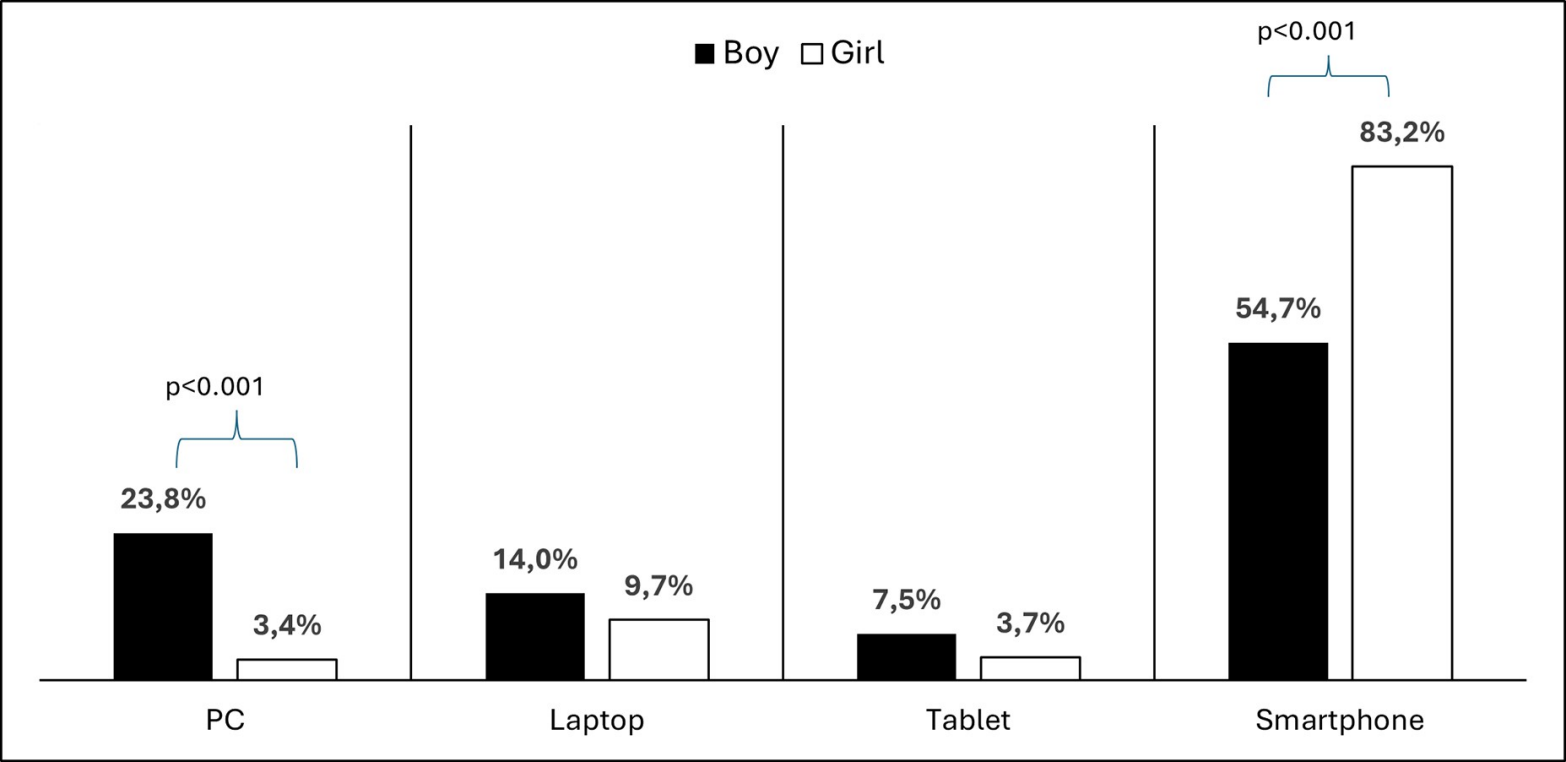
Digital device preferences by sex.

Smartphones were found to be the most preferred digital device in both genders and all age groups (71.3%). Boys were significantly more likely than girls to use desktops (p<0.001), while girls were significantly more likely to use smartphones (X^2^ = 63.443, df = 3, p<0.001, Fig 1). Digital device preference was not significantly related to either the PSQ or the DES percentage (Table 1).

**Fig 2:**
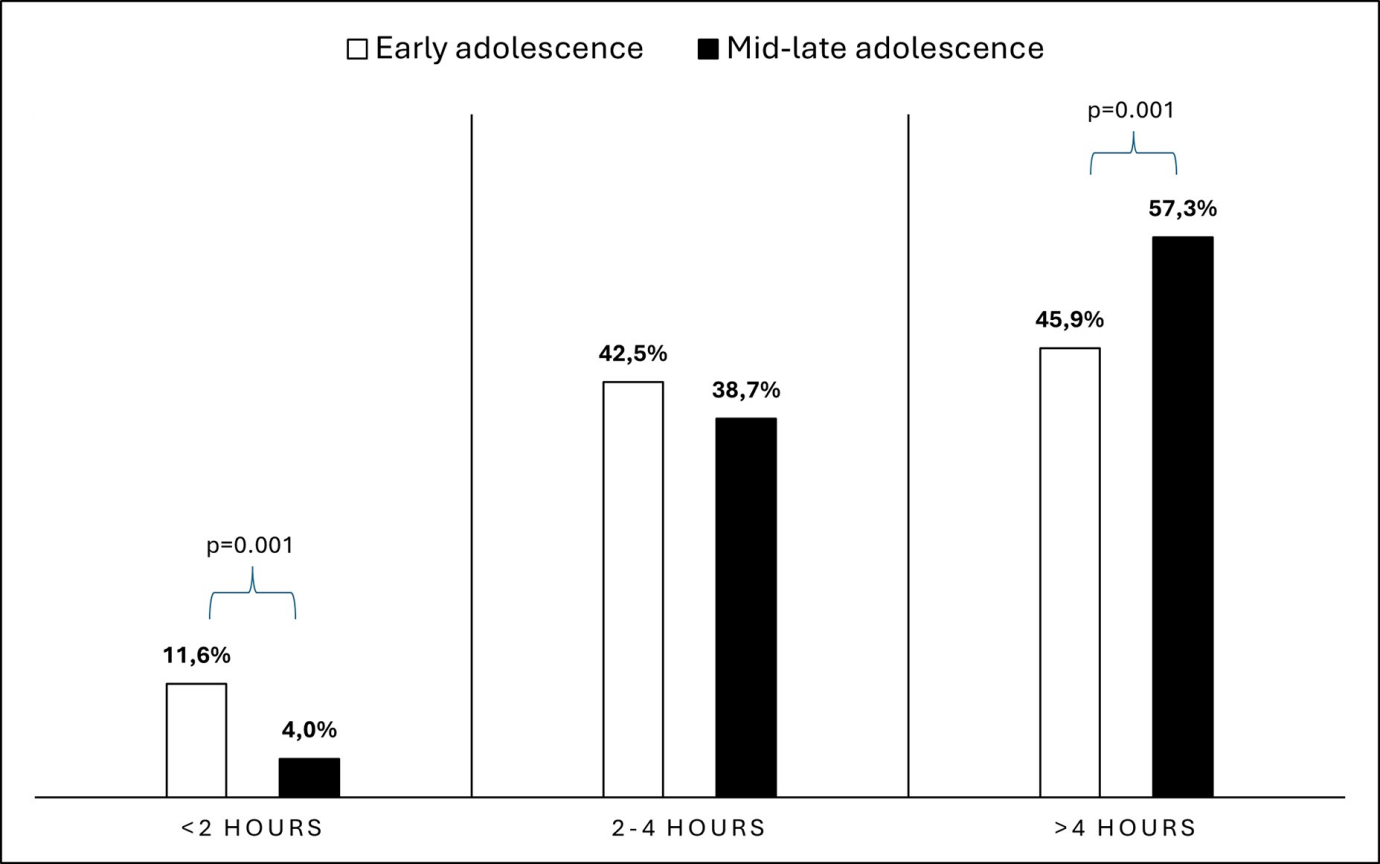
Daily digital media usage by age groups.

A majority of the participants (51.6%) reported using digital media for over four hours daily. It was observed that older adolescents tend to use digital media for significantly longer periods (X^2^ = 13.184, df = 2, p = 0.001, Fig 2). The percentage of DES and PSQ was observed to increase significantly with longer digital media use (p<0.001 and p = 0.001, respectively). Break frequency did not show a significant relationship with DES and PSQ. However, shorter break durations of less than 20 minutes were linked to a higher prevalence of DES (p<0.001, Table 1).

Only 19.5% of participants received online education, although the digital media consumption had increased for 72.3% of the participants compared to the pre-pandemic period. This increase was over 1 hour in 61.7% of them, which was associated with an increased DES percentage (p<0.001, Table 1).

**Fig 3:**
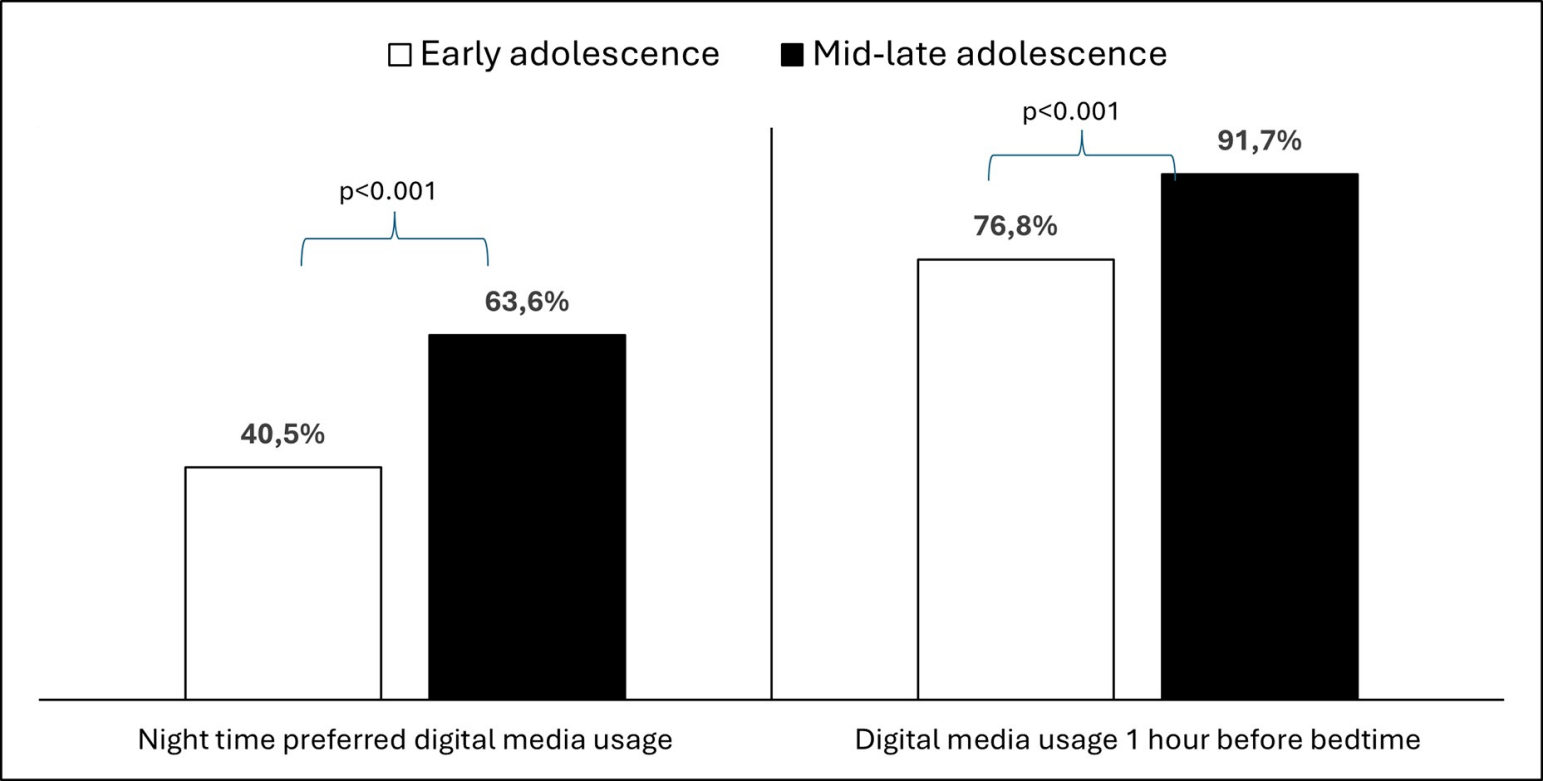
Preferred timing for digital device by age groups.

Despite 52% of participants expressing a preference for nighttime digital media usage, a notable 84.2% admitted to using digital media within the hour leading up to bedtime. Furthermore, it was discovered that older adolescents significantly favor the use of digital media during the night (X^2^ = 27.349, df = 1, p<0.001) and just before sleeping (X^2^ = 21.237, df = 1, p<0.001, Fig 3). Preferring a night-time digital media use was not significantly associated with the percentage of DES but was significantly associated with PSQ (p = 0.001). Digital media use before bedtime was associated with an increase in both DES and PSQ percentage (p<0.001, Table 1).

### Prevalence and risk factors of digital eye strain

**Fig 4:**
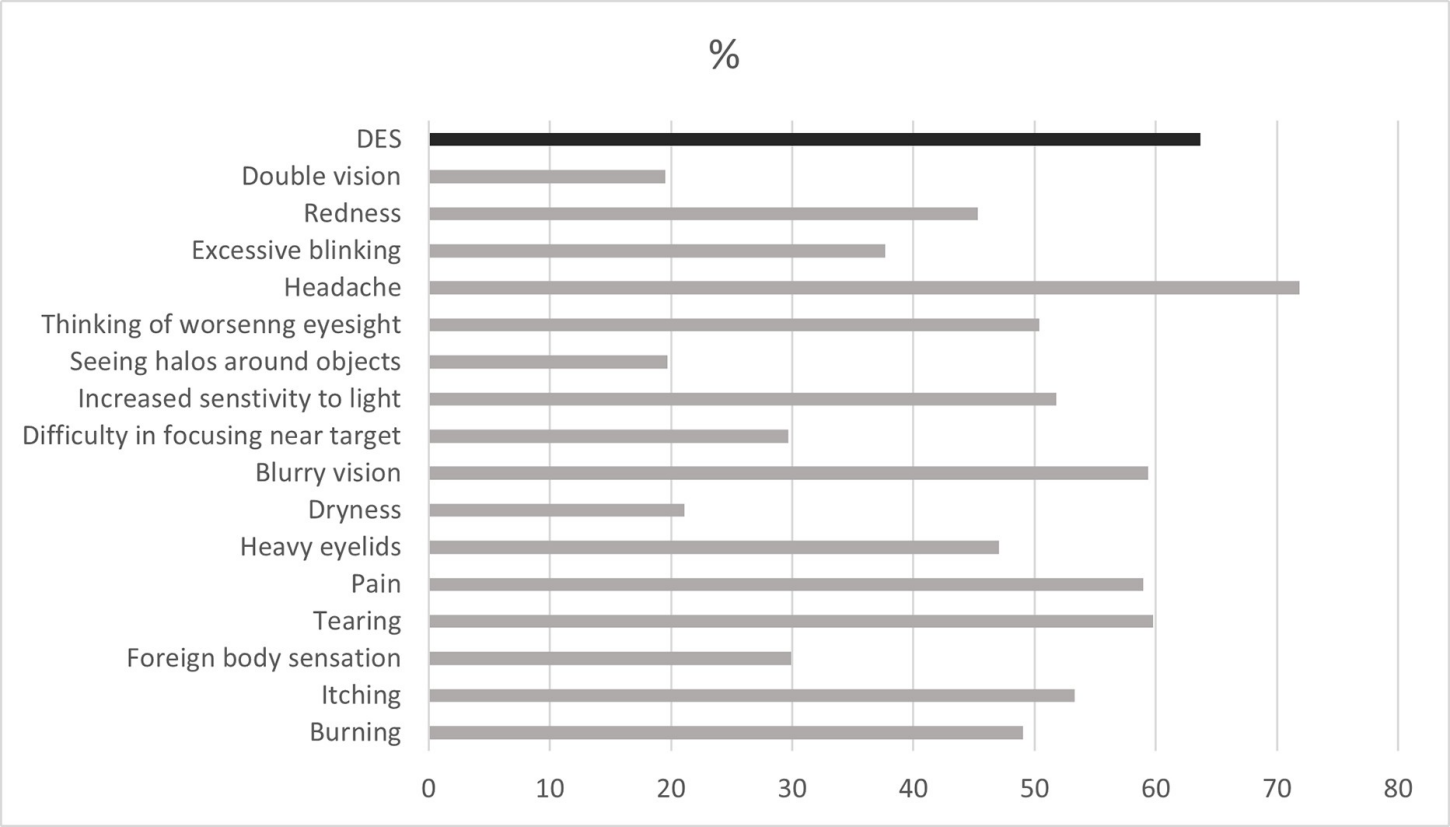
Prevalence and symptoms related to Digital Eye Strain. DES: Digital Eye Strain.

The prevalence of DES was 63.7%. The most common symptoms associated with DES were headache (71.9%), tearing (59.8%), and blurred vision (59.4%), respectively (Fig 4).

To identify the risk factors associated with DES in adolescents, we employed a backward stepwise logistic regression analysis. Out of the seven significant variables identified in pairwise comparisons (age group, sex, daily digital media use, break duration, digital media use one hour before bedtime, digital media consumption has increased since the pandemic, and PSQ), four retained their significance (Table 2).

**Table 2:** Risk factors of Digital Eye Strain.

Variables	B	S.E.	Sig.	Exp(B)	95% C.I. for EXP(B)	
					Lower	Upper
**Daily digital media usage**						
<2 hours			**0.001**			
2–4 hours	1.208	0.399	**0.002**	3.345	1.531	7.308
>4 hours	1.437	0.395	**<0.001**	4.209	1.940	9.132
**Break duration** (ref: >20 min)	0.878	0.230	**<0.001**	2.407	1.532	3.780
**Increased digital media consumption after the pandemic** (ref: no)	0.666	.221	**.003**	1.946	1.262	3.000
**Poor sleep quality** (ref: good)	1.415	.211	**<0.001**	4.118	2.721	6.232
Constant	-2.425	0.438	**<0.001**	0.088		

Ref: reference value

*Note*: Table based on stepwise LR logistic regression analysis data. Bold numbers indicate the statistically significant differences.

Compared to adolescents who use digital media for less than two hours a day, those who use it for 2–4 hours were 3.4 times more likely to experience DES, and those who use it for more than 4 hours are 4.2 times more likely. Similarly, adolescents who reported an increase in digital media use during the pandemic were twice as likely to experience DES. Moreover, adolescents who took breaks of less than 20 minutes were 2.4 times more likely to experience DES. Interestingly, adolescents with PSQ were 4.1 times more likely to experience DES.

### Prevalence and risk factors for poor sleep quality

The mean (SD) global PSQI score calculated for the participants was 5.14 (2.90). The prevalence of PSQ in adolescents was 51.2%.

In order to reassess the factors that were found to be significantly associated PSQ in pairwise comparisons (age group, sex, daily digital media use, preferred timing for digital media use, digital media use one hour before bedtime, online education, and DES) as shown in Table 1, we employed a stepwise logistic regression analysis. The analysis revealed that older age (*Exp(B)* = 2.6), female sex (*Exp(B)* = 2.5), daily digital media exceeding 4 hours (*Exp(B)* = 1.6), participation in online education (*Exp(B)* = 1.8), use of digital media one hour before bedtime (*Exp(B)* = 1.6), and DES (*Exp(B)* = 3.3) were all significantly related to PSQ, as detailed in Table 3.

**Table 3:** Risk factors of poor sleep quality.

Variables	B	S.E.	Sig.	Exp(B)	95% C.I. for EXP(B)	
					Lower	Upper
**Age** (ref: early adolescence)	0.949	0.204	**<0.001**	2.582	1.731	3.853
**Sex** (ref: girl)	-0.903	0.203	**<0.001**	0.405	0.272	0.604
**Daily digital media usage** (ref: <4 hours)^a^	0.479	0.200	**0.017**	1.614	1.090	2.391
**Online education** (ref: no)	0.570	0.257	**0.027**	1.769	1.068	2.929
**Digital media usage 1 hour before bedtime** (ref: no)	0.478	0.204	**0.019**	1.614	1.082	2.407
**Digital eye strain** (ref: no)	1.197	0.210	**<0.001**	3.310	2.194	4.992
Constant	-1.410	0.247	**<0.001**	0.244		

Ref: reference value

^a^: Daily screen time is recorded as a binary variable, indicating less than 4 hours or 4 hours or more, due to its significance in pairwise comparisons.

*Note*: Table based on stepwise LR logistic regression analysis data. Bold numbers indicate the statistically significant differences.

## Discussion

While embracing the advantages of an increasingly digitalized world, we must equally consider the rise in DES prevalence induced by excessive screen use. Our study found a high prevalence of DES among adolescents, specifically 63.7%. This is significantly higher than pre-pandemic levels (24.7%) [31] and supports similar findings from other studies conducted during the pandemic (50–65%) [19, 32, 33]. Furthermore, 61.7% of participants reported that their digital media consumption had increased by >1 hour since the pandemic began in our study. This highlights the concerning prevalence of DES among adolescents, even when e-learning participation was relatively low (19.5%) in our sample. This suggests that the pandemic’s impact on lifestyle extends beyond the immediate period of remote learning, potentially due to an increased reliance on digital technologies. Notably, our data was collected after the peak of the pandemic, indicating a lasting shift towards digital habits that may be contributing to the high prevalence of DES. This is similar to Bhattacharya et al.’s [34] conclusion in their recent review, which referred to it as a ’shadow pandemic’.

In examining specific patterns of digital device use and their associated symptoms, our findings revealed that smartphones were the most preferred digital device, with headaches emerging as the most prevalent DES symptom. These findings are consistent with previous research that has linked headache, eye redness, eye fatigue, and blurred vision to the use of smartphones, computers, and tablets [19, 20]. The impact of increased screen time is particularly evident in recent studies, which have noted a significant increase in average daily screen time among children and adolescents, rising up to 7.02 hours [19], a stark increase from the previously reported 3.9 hours [33]. This surge in screen usage has been linked to a higher prevalence of DES, particularly among children who use digital devices for more than four hours per day [19, 35]. Our study confirms these findings, as 51.6% of participants reported using digital media for over four hours daily, and this was significantly associated with DES.

Given the high prevalence of DES and increased screen time, the role of preventive measures becomes particularly important. Even with beneficial use of digital devices, it is crucial to practice good screen hygiene, including taking breaks according to the 20-20-20 rule to reduce eye strain and promote eye relaxation[36]. Our study found a significant correlation between shorter breaks (less than 20 minutes) and an increased incidence of DES among individuals who took breaks. This highlights the critical role of break duration in the management of DES symptoms. Interestingly, while previous studies have associated DES symptoms with less frequent breaks from screen use [15, 37, 38], our study did not find break frequency to be a determining factor. This discrepancy may be due to the subjective nature of participants’ self-reporting of break habits and duration. Therefore, the length of breaks may be more critical than the frequency of breaks in preventing DES.

Beyond eye health, our investigation revealed significant implications for adolescent sleep patterns. Behavioral and environmental factors, such as digital media use, may reduce adolescent sleep duration with age, rather than biological factors [39]. We found that 51.2% of the study sample had PSQ, with several key risk factors identified: female gender, older age, DES, digital media use before bedtime, online education and higher daily digital media use. These findings align with previous research that has linked the use of electronic devices such as computers, cell phones, and video games before bedtime to morning fatigue and shorter sleep duration [10]. Additionally, overuse of electronic devices during both daytime and bedtime has been linked to short sleep duration, delayed sleep onset latency, and sleep deficiency [11, 40]. The mechanism behind these effects may be partially explained by the blue light emitted by digital screens, which was known to disrupt sleep patterns and melatonin production, while also potentially exacerbating DES [8, 9].

The gender differences in sleep quality patterns revealed by our study deserve particular attention. Studies examining sleep quality in adolescents have consistently reported a higher incidence of PSQ in females relative to males [41, 42]. This observed gender difference in sleep disturbances can be attributed to a variety of factors, including inconsistent sleep schedules, extended durations in bed, and potentially, increased exposure to screen-based and digital devices among females. Moreover, age appears to play a significant role, as older adolescents are more likely to experience sleep disturbances, maintain later bedtimes, and engage in digital activities during evening hours compared to their younger counterparts [43, 44]. In line with these findings, our study identified older adolescents and female gender as risk factors for PSQ. These associations may be driven by the heightened usage of smartphones among female adolescents and the escalated engagement with digital media as adolescents age.

The context of the pandemic provided crucial insights into these patterns of digital media use and their health impacts. The pandemic has been widely recognized as a catalyst for increased screen time, which had negative effects on the sleep and eye health of adolescents [34, 45]. Our findings indicate that adolescents who reported increased digital media consumption after the pandemic were at a higher risk of developing DES, even after accounting for variables related to digital media usage. This suggests that the relationship between pandemic-related changes and health outcomes extends beyond simple increases in screen time, potentially reflecting broader changes in digital media usage habits. Similarly, the association between online education and PSQ might be explained by the evening-focused online course delivery, potentially leading to sleep disruption.

A particularly significant contribution of our research lies in the exploration of the relationship between PSQ and DES, conditions that share significant risk factors, particularly prolonged digital media use. Our analysis revealed a striking bidirectional association between these conditions: adolescents experiencing PSQ were 4.1 times more likely to develop DES, while those with DES were 3.3 times more susceptible to PSQ. This reciprocal relationship underscores a critical comorbidity between the two conditions. To the best of our knowledge, our study represents the first focused investigation into this specific topic, highlighting the need for further research to explore the causal mechanisms underlying this relationship.

While our findings provide valuable insights into the relationships between digital media use, DES, and sleep quality, several limitations warrant consideration. The cross-sectional design limits our ability to establish causal relationships or determine the directionality of observed associations, while our reliance on self-reported questionnaires introduces potential bias due to recall inaccuracies or subjective interpretations. Additionally, our use of categorical variables for screen time may not fully capture the nuanced patterns of digital media use; future research should employ continuous variables and more detailed measures, including frequency and duration of breaks, to provide deeper insights into these associations [4]. Our study also did not account for important environmental factors such as room lighting conditions and screen-to-eye distance, which can significantly affect visual comfort and melatonin suppression [8, 46]. These factors, along with unaccounted-for variables like emotional or behavioral problems, may confound both digital media use and sleep. Notably, our sample consisted of adolescents who applied to the ophthalmology clinic, potentially limiting the generalizability of our results to the wider population. Despite these limitations, the study’s strengths include a large sample size, a focus on adolescents, and its post-pandemic context, providing valuable insights into current digital media use patterns and their implications for adolescent health.

## Conclusions

In light of these compelling results, our findings indicate that limiting adolescent screen time to 4 hours or less per day is crucial for health. Specifically, it results in a 4-fold increase in DES frequency and a 1.6-fold increase in PSQ compared to the suggested healthy threshold. We also identified DES and PSQ as significant comorbidities, necessitating mutual screening. Importantly, good knowledge of DES was associated with decreased odds of DES itself in a recent meta-analysis [4]. This suggests that educational programs focused on early identification and management of DES may be a valuable strategy to improve adolescents’ well-being in the digital age. Public health initiatives must target screen time reduction, encourage healthy habits, and provide collaborative ocular and psychological healthcare services to promote youth well-being.
